# Seven New Pleosporalean Species from Marine Sediment and Seawater

**DOI:** 10.3390/jof12080623

**Published:** 2026-08-19

**Authors:** Wangying Mo, Peisi Wu, Senouwa Segla Koffi Dossou, Mengmeng Wang, Wei Li

**Affiliations:** 1 Guangdong Provincial Key Laboratory of Marine Biotechnology, Shantou University, Shantou 515000, China; 23wymo@stu.edu.cn (W.M.); 23pswu@stu.edu.cn (P.W.); senouwa@stu.edu.cn (S.S.K.D.); 2 Guangdong Engineering Technology Research Center of Offshore Environmental Pollution Control, Shantou University, Shantou 515063, China; 3 Shantou Key Laboratory of Marine Microbial Resources and Interactions with Environment, Shantou University, Shantou 515000, China

**Keywords:** new species, marine fungi, mycoplankton, *Didymosphaeriaceae*, *Sporormiaceae*

## Abstract

The *Pleosporales* order is the largest in the *Ascomycota* phylum and is distributed worldwide, with a wide range of hosts and habitats. Members of this order are closely associated with the aquatic environment, including marine habitats. Many species are isolated from substrates such as mangroves, marine driftwood and seagrass. This study describes seven new species spanning three genera in two families: *Montagnula minispora*, *Paraconiothyrium ellipsosporum*, *P. microconidium*, *P. qingdaoense*, *Westerdykella fulva, W.* *microcarpa*, and *W. sedimenticola*. These new species can be differentiated morphologically from their close relatives by the presence of an anamorph, the type of sporangium and the color, shape and size of the conidia. The LSU-ITS-tef1-rpb2 phylogeny revealed seven independent and highly supported clades, providing important evidence for the introduction of these new species. Ecologically, they were retrieved from the marine environment, spanning the intertidal, coastal and deep-sea zones, thereby expanding our knowledge of the distribution and species diversity of pleosporalean fungi.

## 1. Introduction

The genera *Montagnula*, *Paraconiothyrium*, and *Westerdykella* are important members of the *Pleosporales* (*Dothideomycetes*). The genus *Montagnula* was established by Berlese in 1896, with *M. infernalis* named as the type species [1]. It is characterized by globose, clavate bitunicate asci, immersed ascomata with a clypeus, and fusoid to ellipsoid ascospores with longitudinal and transverse septa [1,2]. *Paraconiothyrium* was introduced by Verkley to accommodate coniothyrium-like asexual morphs, with the type species being *P. estuarinum* [3]. Its asexual morph is characterized by eustromatic or pycnidial conidiomata, phialidic conidiogenous cells, aseptate to one-septate, and hyaline to brown conidia [3,4]. Meanwhile the genus *Westerdykella* was first described by Stolk in 1955, with *W. ornata* as the type [5]. It is predominantly known for its sexual morph, which produces cleistothecial ascomata and dark, multi-celled ascospores. However, some species also produce a pycnidial asexual morph [5,6]. Multi-locus phylogenetic analyses (e.g., LSU, SSU, ITS and tef1) have led to a taxonomic revision of all three genera, which are currently placed within the families *Didymosphaeriaceae* (*Montagnula* and *Paraconiothyrium*) and *Sporormiaceae* (*Westerdykella*) [7].

Ecologically, species of *Montagnula*, *Paraconiothyrium*, and *Westerdykella* exhibit diverse life strategies, acting as saprobes, parasites, or endophytes on a wide range of substrates [3,7]. *Montagnula* species are primarily saprobic on dead wood, stems, branches, bark, and leaves. They have a cosmopolitan distribution, having been recorded in at least 29 countries across Asia, Europe, North America, and Australia. They are notably associated with the *Poaceae* family [2]. *Paraconiothyrium* species predominantly act as saprobes on dead plant materials and have a global distribution across both temperate and tropical regions. They have been reported on hosts, including members of the *Fabaceae*, *Poaceae*, and *Rosaceae* families [3,8]. *Westerdykella* species are frequently isolated from soil and sediment, including hypersaline and agricultural environments, with existing records from countries such as Iran, Japan, Spain, and Portugal [9,10]. These three genera, therefore, occupy a range of terrestrial habitats, from decaying plant matter to soil, contributing to nutrient cycling and organic matter decomposition.

The species diversity of these genera in marine and hypersaline environments remains relatively unexplored. *Westerdykella* has been reported more frequently from such habitats, with species such as *W. ornata* being isolated from mangrove mud in Mozambique and *W. dispersa* from marine sediments. The latter is well known for its production of bioactive compounds and its ability to biosynthesize iron nanoparticles [5,9,11]. A recent study from hypersaline lagoons in Spain described two new *Montagnula* species (*M. globospora* and *M. terricola*) and reclassified *Herpotrichia striatispora* as *M. striatispora*, highlighting the potential for novel taxa in saline environments [9]. *Paraconiothyrium* has also been found in estuarine and marine settings. For example, *P. salinum* was described from marine macroalgae in Portugal [12]. The discovery of novel species in lagoonal sediments, macroalgae, and intertidal zones suggests that these genera harbor significant hidden diversity in marine and saline ecosystems, emphasizing the need for further targeted surveys.

During an investigation of culturable fungi from marine environments, a number of pleosporalean isolates were discovered in the intertidal and coastal zones of China, as well as in the deep-sea regions of the Western Pacific Ocean. Using morphology and multi-locus phylogeny methods, these isolates were identified as seven new species belonging to three genera within two families. The aims of this study are: (1) to systematically identify and clarify the phylogenetic relationships of these fungi species, and (2) to characterize and introduce the new species.

## 2. Materials and Methods

### 2.1. Sample Collection and Fungal Isolation

Seawater and sediment samples were collected from the intertidal zones of Guangdong, Jiangsu, Liaoning, Shandong, and Zhejiang provinces. Three liters of seawater samples were collected from each site into a sterilized sampling bottle and filtered through 0.45 µm pore-size Sterivex membranes (Millipore, Billerica, MA, USA). For intertidal sediment samples, 200 g of sediment was collected from each site into a sterilized sampling bag. Coastal and deep-sea sediment samples were collected from the Yellow Sea (China) and the Western Pacific Ocean, respectively. When sampling, surficial sediment samples (0–20 cm) were collected using a 0.05 m^3^ stainless steel grab sampler (Wildco, Saginaw, MI, USA), All samples were stored in a freezer at 4 °C before being taken back to the laboratory as soon as possible.

Fungi were isolated from the seawater using the membrane patch method. The seawater membrane was cut into segments (ca. 5 × 5 mm) and placed onto the isolation medium. Fungi were isolated from sediments using the dilution-plate method [13] and the direct-isolation method, with some modifications. Relative to the dilution-plate method, 10 g of each sediment was suspended in 90 mL of sterile seawater in a 100 mL triangular glass flask and thoroughly mixed by shaking at 150 rpm at 25 °C for 20 min. The suspension was then diluted to a series of four concentrations (10^−1^, 10^−2^, 10^−3^, and 10^−4^) and plated onto five types of isolation medium, with three replicates. For the direct-isolation method, approximately 1 g of each sediment sample was spread directly onto the five types of isolation medium, with three replicates. The five types of isolation media include: (1) Martin medium (MM): each 1 L of medium contains peptone 5.0 g, dextrose 10.0 g, monopotassium phosphate 1.0 g, magnesium sulfate 0.5 g, chloramphenicol 0.1 g, rose Bengal 0.033 g, agar 20.0 g, and seawater 1 L; (2) 1/10 potato dextrose agar (1/10 PDA): each 1 L of medium contains potato 20.0 g, dextrose 2.0 g, agar 20.0 g, and seawater 1 L; (3) 1/5 malt extract agar (1/5 MEA): each 1 L medium contains malt extract 4.0 g, peptone 0.2 g, dextrose 4.0 g, agar 20.0 g, and seawater 1 L; (4) Corn meal agar (CMA): each 1 L medium contains corn meal 30.0 g, agar 20.0 g, and seawater 1 L; and (5) Yeast extract peptone glucose agar (YPG): each 1 L medium contains yeast extract 1.25 g, peptone 1.25 g, dextrose 4.0 g, agar 20.0 g, and seawater 1 L. For controlling contamination, ampicillin (500 mg/L) and streptomycin (500 mg/L) were selected and used for fungal isolation in this study.

All plates were incubated at room temperature and examined for fungal hyphae every two days using an Olympus SZX7 stereomicroscope (Tokyo, Japan). Individual colonies were picked up with a sterilized needle and transferred to fresh PDA plates on the benchtop. After seven days incubation at room temperature, all the cultures were purified using an optimized protocol for single-spore isolation [14]. All isolates examined in this study were deposited in Wei Li’s personal culture collection (WL). Type specimens of new species were deposited in the Fungarium of the Institute of Microbiology (HMAS), with the ex-type living cultures deposited in the China General Microbiological Culture Collection Center (CGMCC).

### 2.2. Morphological Observation

The isolates were incubated on PDA, oatmeal agar (OA), and MEA. After 7 days of incubation in the dark, the characteristics of the cultures were observed, including colony morphology, pigmentation, and odor. Colors were assessed according to Kornerup & Wanscher’s color charts [15]. Micromorphological characteristics were examined and photographed using water as a mounting medium under an Olympus BX53 microscope (Tokyo, Japan) with differential interference contrast (DIC) optics. For each species, 30 conidiogenous cells and 50 conidia were randomly selected for mounting and measurement.

### 2.3. DNA Extraction and Amplification

Genomic DNA was extracted from fungal mycelia using a modified CTAB protocol as described by Guo et al. [16]. Four loci, including partial large-subunit ribosomal RNA (LSU), 5.8S nuclear ribosomal RNA gene with the two flanking internal transcribed spacer (ITS), partial translation elongation factor (tef1), and partial RNA polymerase second-largest subunit (rpb2) gene regions, were amplified using primer pairs LR0R/LR5 [17], ITS5/ITS4 [18], EF1-983F/EF1-2218R [19], and RPB2-5f2/RPB2-7cR [20,21], respectively. Amplification reactions were performed in a 25 μL volume containing 12.5 μL of 2 × Taq PCR Master Mix (Vazyme Biotech Co., Ltd., Nanjing, China), 1 μL of each 10 μM primers, and 1 μL of the undiluted genomic DNA. The final volume was adjusted with distilled and deionized water, and then place it into the PCR amplification instrument (Dongsheng, EDC-810, Beijing, China) for the reaction. The PCR conditions were set as follows: 95 °C for 5 min, followed by 38 cycles of 95 °C for 30 s, annealing at 50 °C (for LSU)/54 °C (for ITS)/57 °C (for tef1 and rpb2) for 30 s, and extension at 72 °C for 30 s, with a final elongation step at 72 °C for 10 min. The PCR products were visualized on a 1% agarose electrophoresis gel. Sequencing was performed bidirectionally by the BGI Write Company (Beijing, China). Consensus sequences were obtained using SeqMan of the Lasergene software package v. 14.1 (DNAstar, Madison, WI, USA).

### 2.4. Phylogenetic Analyses

The sequences of the pleosporalean strains examined in this study, along with the reference strains, are listed in Table 1. For each locus, the sequences were aligned using MAFFT v. 7 [22], with manual adjustments made where necessary. The best-fitting nucleotide substitution models according to the Akaike Information Criterion (AIC) were selected using jModelTest v. 2.1.7 [23,24]. The alignments derived from this study have been deposited in TreeBASE, and the taxonomic novelties have been deposited in FungalNames.

Phylogenetic analyses of the combined dataset were performed using Bayesian infer-ence (BI) and maximum-likelihood (ML) methods. The BI analyses were conducted using MrBayes v. 3.2.1 [25], following the protocol of Wang et al. [26], with optimization of each locus treated as a partition in the combined analyses. This was based on the Markov Chain Monte Carlo (MCMC) approach [27]. All characters were given equal weighting, and gaps were treated as missing data. Stationarity of the analyses was determined by examining the standard deviation of split frequencies (<0.01) and –ln likelihood plots in AWTY [28]. The ML analyses were conducted using PhyML v. 3.0 [29], with 1000 bootstrap replicates. The general time-reversible model was applied with an invariable gamma-distributed rate variation (*GTR+I+G*).

## 3. Results

### 3.1. Phylogeny

**Figure 1:**
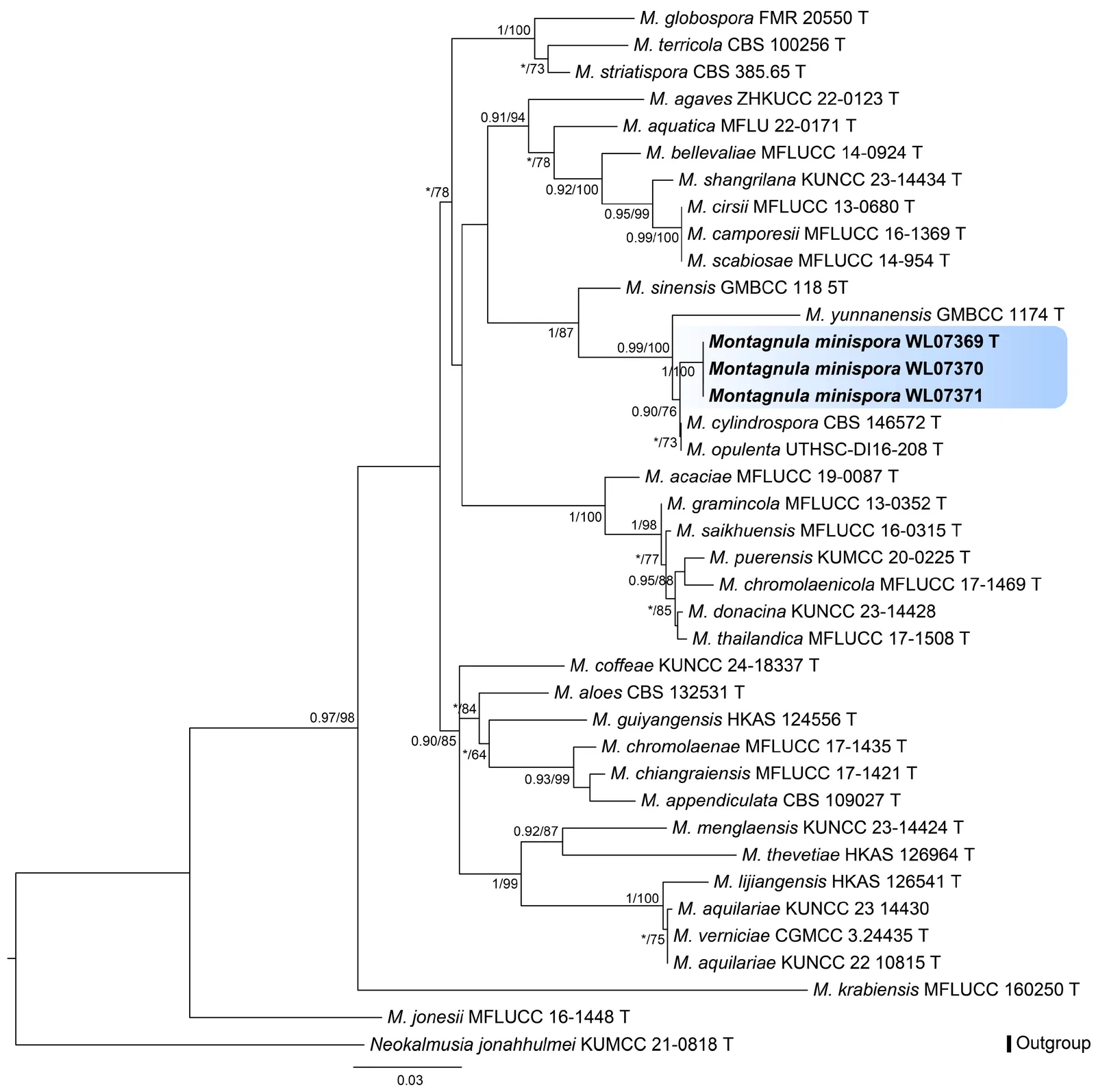
The phylogenetic relationships among species within the genus *Montagnula*. The fifty-percent majority rule was adopted, and the consensus tree was generated using a Bayesian analysis based on a four-locus combined dataset (LSU-ITS-tef1-rpb2). The Bayesian posterior probabilities (PP > 0.9) and PhyML bootstrap support values (BS > 70%) are displayed at the nodes (PP/BS), as are “*” to indicate PP < 0.9. The tree was rooted to *Neokalmusia jonahhulmei* (KUMCC 21-0818 T). Ex-type cultures are indicated with “T”. New species introduced in this paper are marked in bold.

Phylogenetic analyses of the genus *Montagnula* were conducted by using a combined dataset of LSU (866 bp), ITS (476 bp), tef1 (818 bp), and rpb2 (965 bp). For the BI analysis, the *GTR+I+G* model was selected for the LSU, ITS, and tef1 loci, and the *GTR+G* for the rpb2 locus. The resulting phylogeny showed that our isolates clustered as a single, well-supported clade (PP/BS = 1/100) that was closely related to *M. cylindrospora*, *M. opulenta* and *M. yunnanensis*. This supports the classification of the new species, *M. minispora* (Figure 1).

**Figure 2:**
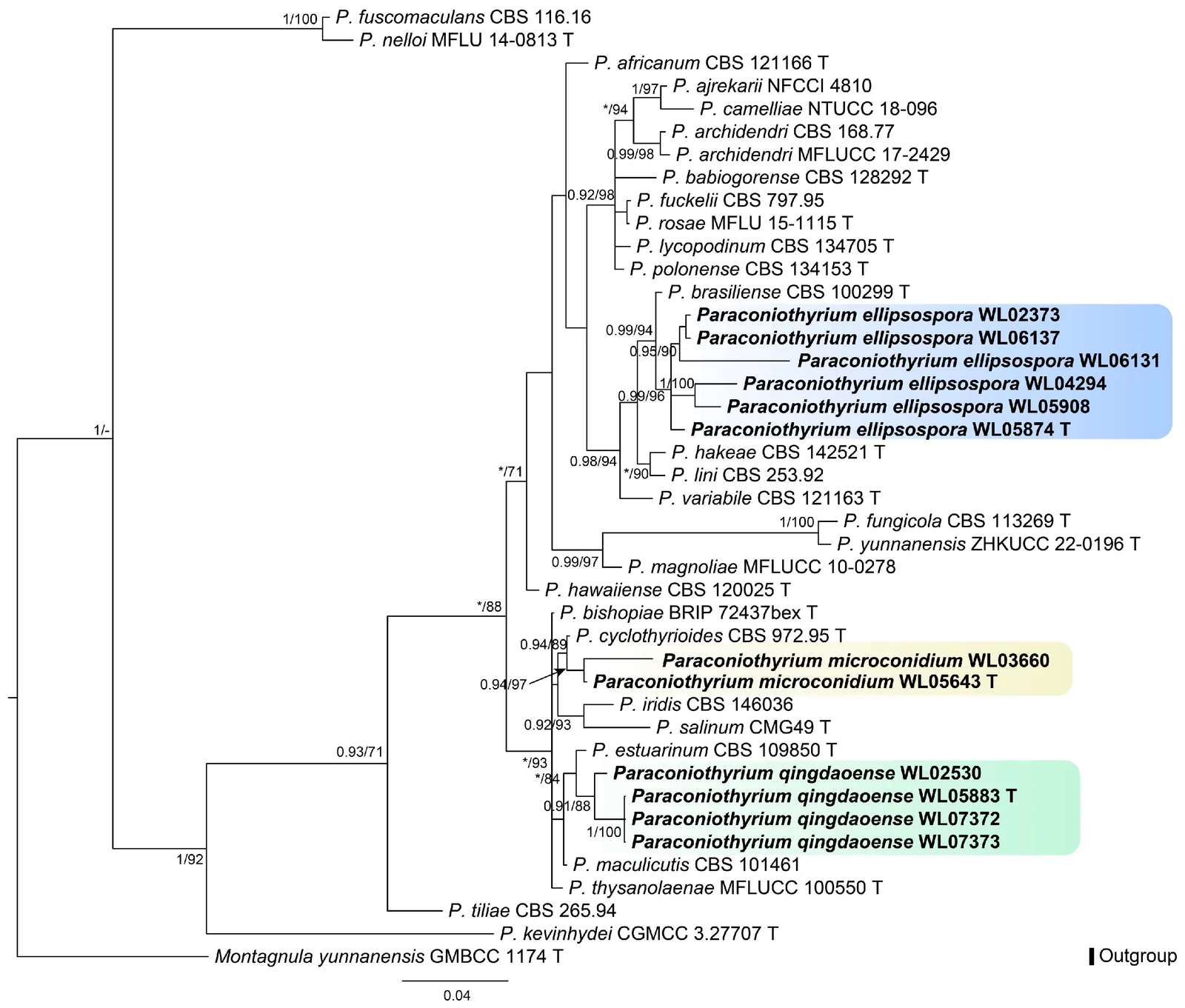
The phylogenetic relationships among species within the genus *Paraconiothyrium*. The fifty-percent majority rule was applied, and the consensus tree was constructed using a Bayesian analysis based on a four-locus combined dataset (LSU-ITS-tef1-rpb2). The Bayesian posterior probabilities (PP > 0.9) and PhyML bootstrap support values (BS > 70%) are displayed at the nodes (PP/BS), as are “*” and “-” to indicate PP < 0.9 and BS < 70%, respectively. The tree was rooted to *Montagnula yunnanensis* (GMBCC 1174 T). Ex-type cultures are indicated with “T”. New species introduced in this paper are marked in bold.

Meanwhile, the combined dataset of LSU (916 bp), ITS (694 bp), tef1 (908 bp) and rpb2 (903 bp) was used for the phylogenetic analyses of genus *Paraconiothyrium*. For the BI analysis, the *GTR+I* model, *GTR+G* model, *GTR+I+G* model, and SYM+G model were selected for the locus LSU, ITS, tef1, and rpb2, respectively. As illustrated in Figure 2, our isolates were clustered into three well-supported clades, indicating three new species, namely *P. ellipsosporum*, *P. microconidium*, and *P. qingdaoense*.

**Figure 3:**
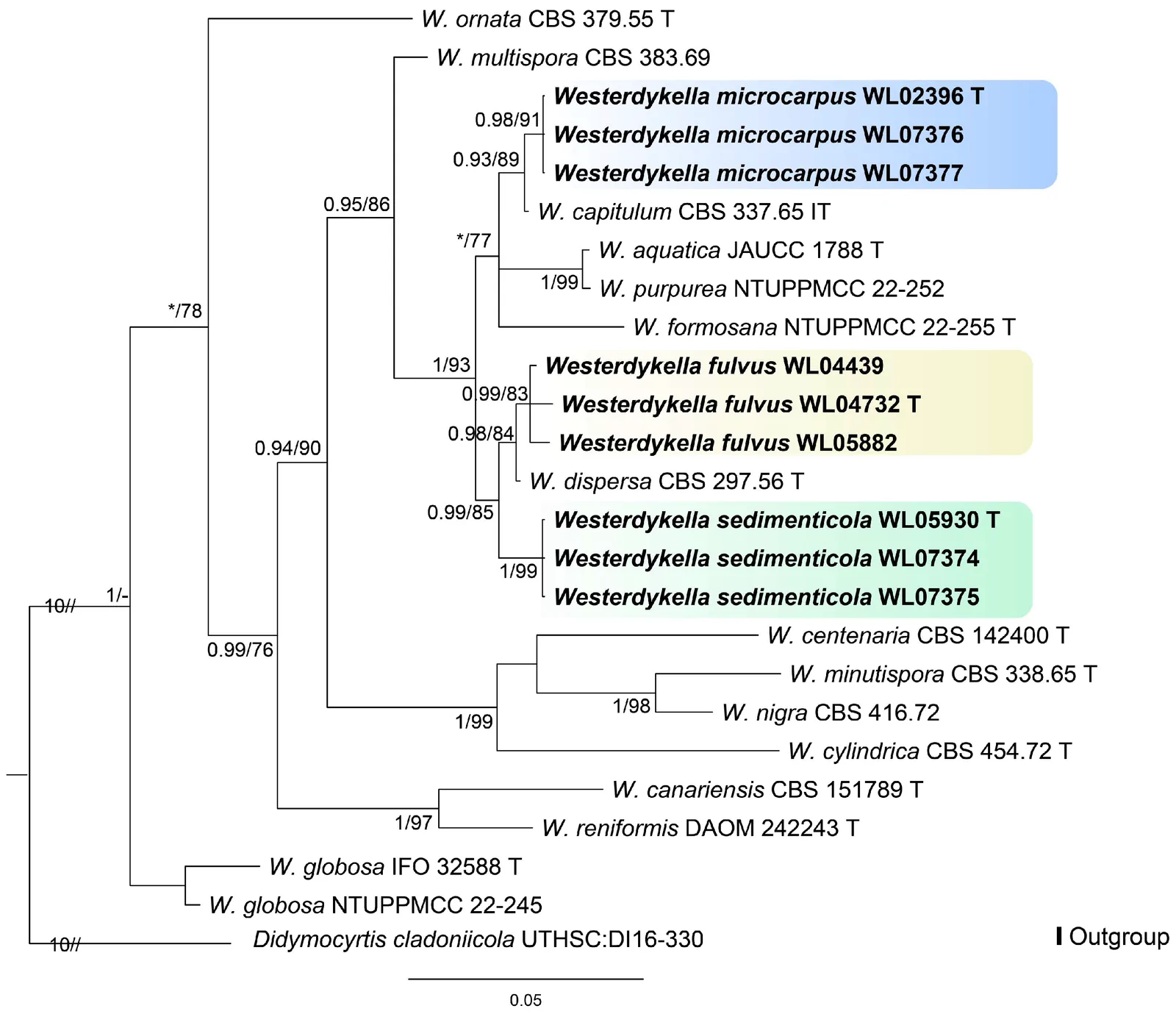
The phylogenetic relationships among species within the genus *Westerdykella*. The fifty-percent majority rule was applied and the consensus tree was generated using a Bayesian analysis based on a four-locus combined dataset (LSU-ITS-tef1-rpb2). The Bayesian posterior probabilities (PP > 0.9) and PhyML bootstrap support values (BS > 70%) are displayed at the nodes (PP/BS), as are “*” and “-” to indicate PP < 0.9 and BS < 70%, respectively. The tree was rooted to *Didymocyrtis cladoniicola* (UTHSC:DI16-330). Ex-type cultures are indicated with “T”, and ex-isotype with “IT”. New species introduced in this paper are marked in bold.

Similarly, the phylogenetic analyses of the genus *Westerdykella* were conducted using a combined dataset of LSU (860 bp), ITS (518 bp), tef1 (832 bp) and rpb2 (967 bp). For the BI analysis, the *GTR+I+G* model was selected for the LSU, ITS and tef1 loci, and the *GTR+G* model was chosen for the rpb2 locus. The results showed that our isolates formed three separated and well-supported clades, indicating three new species, namely *W. fulva*, *W. microcarpa* and *W. sedimenticola* (Figure 3).

### 3.2. Taxonomy

*Ascomycota* Caval.-Sm., Biol. Rev. 73: 247 (1998)

*Dothideomycetes* O.E. Erikss. & Winka, Myconet 1(1): 5 (1997)

*Pleosporales* Luttr. ex M.E. Barr, Prodr. Cl. Loculoasc. (Amherst): 67 (1987)

*Didymosphaeriaceae* Munk, Dansk bot. Ark. 15(no. 2): 128 (1953)

*Montagnula* Berl., Icon. fung. (Abellini) 2(2–3): 68 (1896)

**Figure 4:**
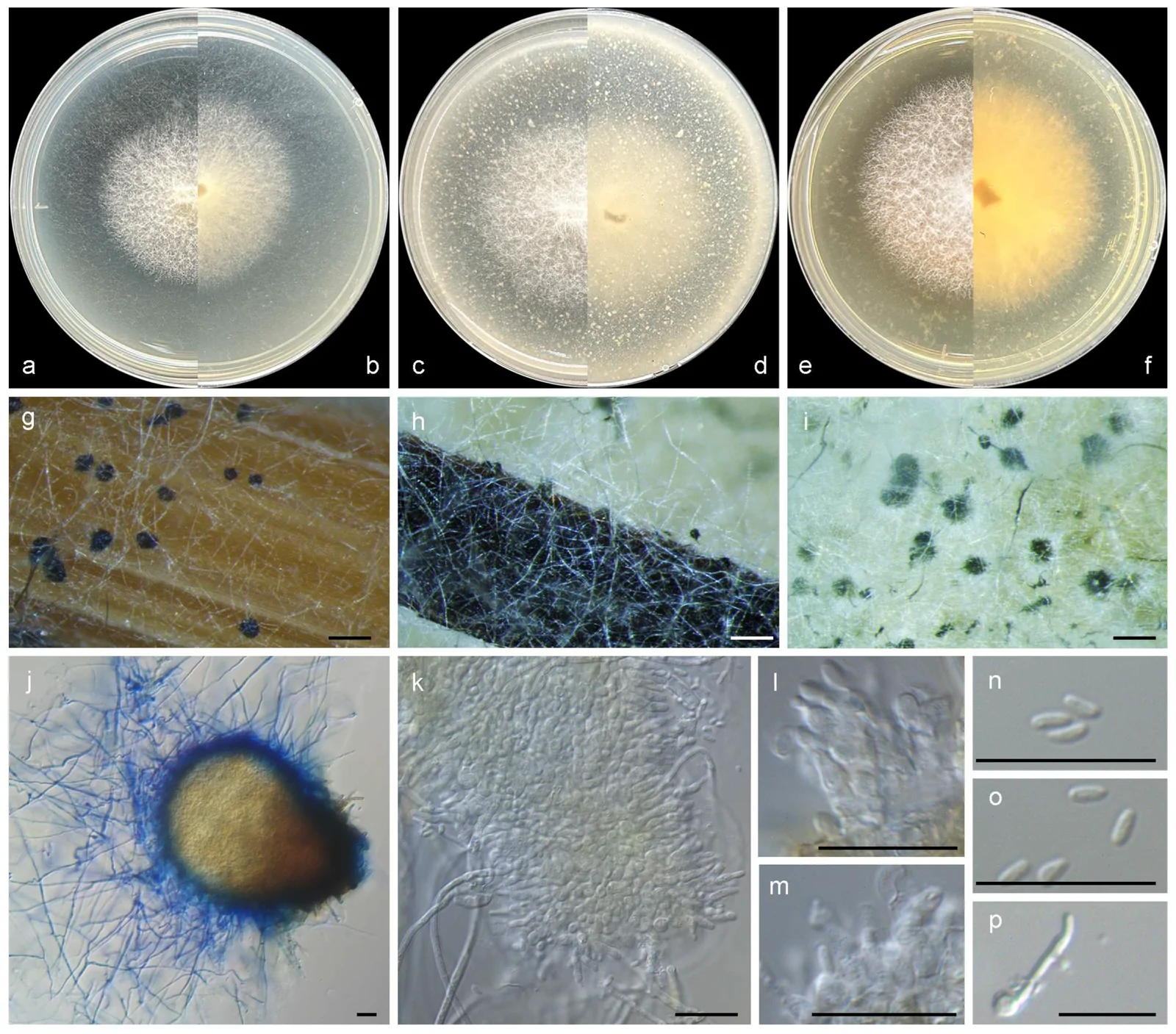
Morphological characteristics of *Montagnula minispora* (from ex-type WL07369). (**a**–**f**): Colony morphology (obverse and reverse) on PDA, OA, and MEA after 7 days of cultivation at 25 °C. (**g**–**j**): Pycnidia. (**k**–**m**): Conidiogenous cells. (**n**,**o**): Conidia. (**p**): Germinated spore. Scale bars: (**g**–**i**) = 500 μm, (**j**–**p**) = 10 μm.

***Montagnula minispora*** M.M. Wang, W.Y. Mo and W. Li sp. nov., Figure 4.

*FungalNames*: FN 573958

*Etymology*: Named after the smaller conidia of the new species.

*Typus*: China, Zhejiang Province, Zhoushan city (122.22° E, 30.32° N), from intertidal sediment of a mudflat, 4 July 2023, W. Li, M.M. Wang, H.H. Ma and S.Y. Yang (HMAS 354888, holotype designated here, dried culture on PDA; culture ex-type CGMCC 3.40482 = WL07369; GenBank Accession No. LSU = PZ764971, ITS = PZ764947, tef1 = PZ775706, rpb2 = PZ775718).

Pycnidia semi-immersed to superficial on mycelial mats or plant tissue, solitary, broadly rounded at the base, tapering towards the apex, asymmetrically tear-shaped, pyriform to obovoid, 80–140 μm diam, dark brown to black, with hyphal appendages on the outer wall; ostiole prominent at maturity; wall composed of several layers of pseudoparenchyma, 5–11 μm thick, outer layers dark brown, inner layers pale yellow. Conidiogenous cells phialidic, ampulliform, cylindrical to lageniform, hyaline, 1.1–2.0 × 3.5–7.7 μm (mean 1.52 × 5.43 μm, *n* = 30), with a distinct apical neck. Conidia hyaline, aseptate, ellipsoidal to long-ellipsoidal, with rounded ends, smooth-walled, 0.8–1.7 × 1.8–4.0 μm (mean 1.23 × 2.86 μm, *n* = 50).

*Culture characteristics*: Colonies on PDA are 32–38 mm diam in 7 d at 25 °C, flat, loose, nearly circular, margin entire, aerial mycelium white to light gray (1A1–1B2); reverse uniformly pale creamy-yellow to pale yellowish (2A3–2A4), margin nearly colorless. Colonies on OA are 38–42 mm diam, velvety, nearly circular, margin undulate; aerial mycelium pure white to milky white (1A1–1A2), denser in the center, sparser at the margin; reverse uniformly pale creamy-yellow to pale grayish-white (2A3–1A2). Colonies on MEA are 40–45 mm diam, cottony, nearly circular, margin entire, aerial mycelium white (1A1); reverse light yellow to orange (2A4–5A6).

*Otherexamined isolates*: China, Zhejiang Province, Zhoushan city (122.22° E, 30.32° N), from intertidal sediment of a mudflat, 4 July 2023, W. Li, M.M. Wang, H.H. Ma and S.Y. Yang (WL07370); ibid. WL07371.

*Notes*: The genus *Montagnula* was established in 1896 to distinguish two dictyosporous species, *M. infernalis* and *M. gigantean*, from *Pleospora* [1]. Currently, around 55 species are accepted in this genus (MycoBank, https://www.mycobank.org/; accessed on 10 July 2026), but only about 35 species have available molecular sequences (shown in Table 1). Most *Montagnula* species, including the type species *M. infernalis*, have exclusively been discovered with a sexual morph, characterized by immersed ascomata under a clypeus, long pedicellate asci, and fusiform, brown ascospores [1,7]. To date, only four species have been found to possess an asexual morph, namely *M. agaves*, *M. cylindrospora*, *M. globospora* and *M. sinensis* [9,30,31]. This study introduces the fifth anamorphic species, *M. minispora*. On the phylogenetic tree based on LSU-ITS-tef1-rpb2 dataset, *M. minispora* was closely related to *M. cylindrospora*, *M. opulenta* and *M. yunnanensis* (Figure 1). Morphologically, the new species can be distinguished by its smaller conidia size (0.8–1.7 × 1.8–4.0 μm in *M. minispora* vs. 1.8–4 × 3–6 μm in *M. cylindrospora*) [32], the absence of a sexual morph (present in *M. opulenta*) [33], and its ability to produce conidia (sterile in artificial culture in *M. yunnanensis*) [32].

*Paraconiothyrium* Verkley, Stud. Mycol. 50(2): 327 (2004)

**Figure 5:**
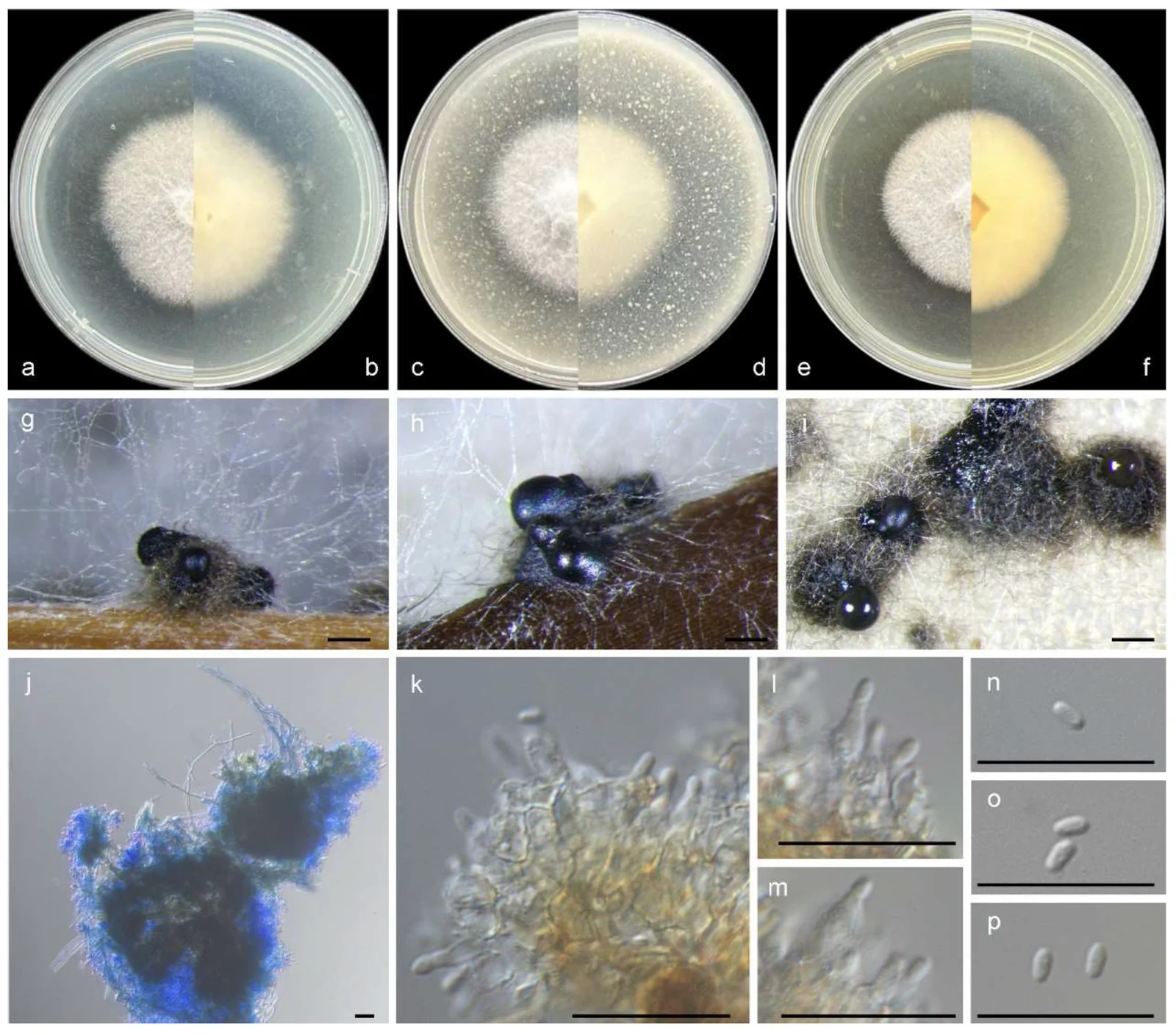
Morphological characteristics of *Paraconiothyrium ellipsosporum* (from ex-type WL05874). (**a**–**f**): Colony morphology (obverse and reverse) on PDA, OA, and MEA after 7 days of cultivation at 25 °C. (**g**–**j**): Pycnidia. (**k**–**m**): Conidiogenous cells. (**n**–**p**): Conidia. Scale bars: (**g**–**i**) = 500 μm, (**j**–**p**) = 10 μm.

***Paraconiothyrium ellipsosporum*** M.M. Wang, W.Y. Mo and W. Li sp. nov., Figure 5.

*FungalNames*: FN 573959

*Etymology*: Named after the ellipsoidal conidia of this new species.

*Typus*: China, Shandong Province, Qingdao city (120.47° E, 36.09° N), from intertidal sediment, 12 March 2021, Y. Zheng, Z.H. Pan and Y.R. Ma (HMAS 354889, holotype designated here, dried culture on PDA; culture ex-type CGMCC 3.40479 = WL05874; GenBank Accession No. LSU = PZ764975, ITS = PZ764952, tef1 = PZ775713, rpb2 = PZ775723).

Pycnidia semi-immersed to superficial on oatmeal agar, solitary or clustered on sterilized toothpicks and pine needles. Mature pycnidia shiny black to charcoal-black, smooth or slightly glossy, hard, and carbonaceous in texture; subglobose, depressed-globose, or irregularly globose, 100–180 μm diam, some with an inconspicuous papillate ostiole. Pycnidial wall 4–6 cells thick, with outer cells dark brown, thick-walled, polygonal, pigmented; inner layers gradually becoming thin-walled and hyaline. Conidiogenous cells phialidic to subcylindrical, hyaline, smooth, 1.4–2.6 × 2.4–4.3 μm (mean 1.97 × 3.26 μm, *n* = 30), with inconspicuous periclinal thickening and collarette, occasionally with a single annellidic proliferation, arising from the pycnidial wall. Conidia ellipsoidal or short-cylindrical, with rounded ends, 1.3–1.9 × 2.0–3.4 μm (mean 1.56 × 2.79 μm, *n* = 50), hyaline when released, becoming pale olive-brown at maturity, thin-walled, smooth.

*Culture characteristics:* Colonies on PDA are 35–40 mm diam in 7 d at 25 °C, cottony, loose, nearly circular, margin entire, aerial mycelium white to light gray (1A1–1B2), slightly raised in the center; transparent to pale yellow exudates present in some areas (2A3); reverse pale yellow to yellowish-brown centrally (2A4–4B5), nearly colorless at the margin, without obvious pigment diffusion. Colonies on OA are 40–45 mm diam, velvety, nearly circular, margin undulate, aerial mycelium white (1A1), denser in the center, sparser at the margin; reverse nearly colorless to light gray (1A1–1B1), without obvious pigment. Colonies on MEA are 48–53 mm diam, circular, loosely filamentous, margin slightly rough; reverse yellowish-brown to brown centrally (5C6–6D7), margin light yellow (2A4), with obvious pigment diffusion.

*Other examined isolates*: China, Liaoning Province, Huludao city (120.79° E, 40.60° N), from intertidal sediment, 9 May 2014, W. Li and X.M. Bian (WL02373); ibid. (120.34° E, 36.06° N), from intertidal seawater, 18 April 2022, M.M. Wang and Y. Zheng (WL06131); ibid. (WL06137); Shandong Province, Weihai city (122.19° E, 37.50° N), from intertidal seawater, 25 November 2020, M.M. Wang and Y. Zheng (WL05908); the Western Pacific Ocean (128.95° E, 11.78° N), from deep-sea sediment, 8 July 2019, C.H. Feng (WL04294).

*Notes*: The genus *Paraconiothyrium* was established in 2004 to encompass coniothyrium-like asexual morphs, including the mycoparasite *Coniothyrium minitans* and four new species (*P. estuarinum* as the type species, *P. brasiliense*, *P. cyclothyrioides*, and *P. fungicola*) [3]. However, subsequent multi-locus phylogenetic analyses revealed the genus to be paraphyletic within the *Didymosphaeriaceae*. This led to some species being transferred to *Paraphaeosphaeria* and new related genera (e.g., *Alloconiothyrium* and *Dendrothyrium*) being described [34]. Currently, about 32 species are recognized within this genus (MycoBank, https://www.mycobank.org/; accessed on 10 July 2026). This study introduces three new species. The first, *P. ellipsosporum*, is phylogenetically closely related to *P. brasiliense*, *P. hakeae*, *P. lini* and *P. variabile* (Figure 2). Morphologically, the new species differs in the shape and size of its conidia, ellipsoidal or short-cylindrical, 1.3–1.9 × 2.0–3.4 μm in *P. ellipsosporum* compared to ellipsoid to short-cylindrical, 2–3.6 × 3.2–5.3 μm in *P. brasiliense*, subcylindrical, 2 × 2.5–4 μm in *P. hakeae*, and subcylindrical to ellipsoidal, 1–2.5 × 2.5–5 µm in *P. variabile* [3,35,36].

**Figure 6:**
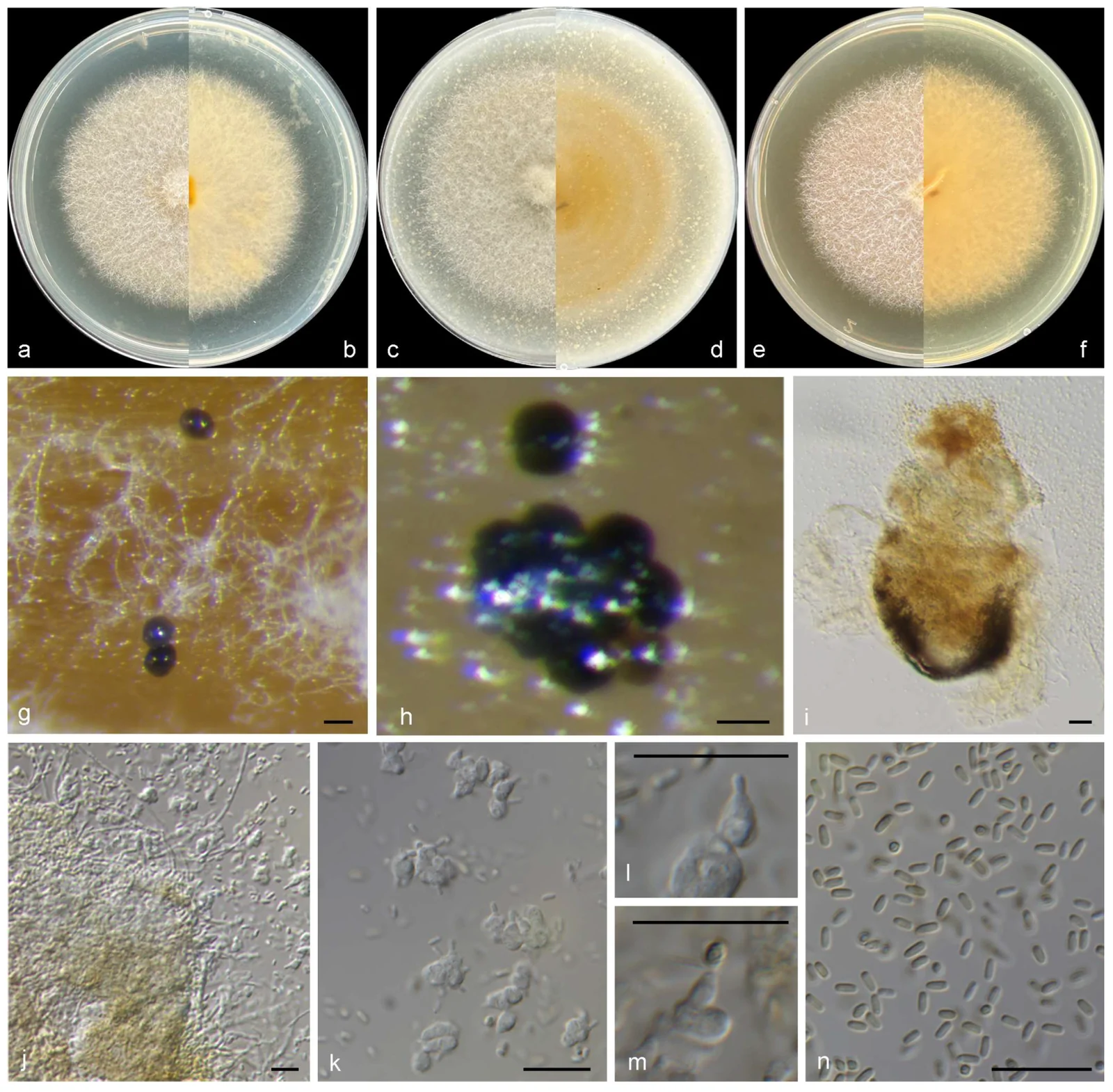
Morphological characteristics of *Paraconiothyrium microconidium* (from ex-type WL05643). (**a**–**f**): Colony morphology (obverse and reverse) on PDA, OA, and MEA after 7 days of cultivation at 25 °C. (**g**–**i**): Pycnidia. (**j**–**n**): Conidiogenous cells and conidia. Scale bars: (**g**,**h**) = 100 μm, (**i**) = 20 μm, (**j**–**n**) = 10 μm.

***Paraconiothyrium microconidium*** M.M. Wang, W.Y. Mo and W. Li sp. nov., Figure 6.

*FungalNames*: FN 573960

*Etymology*: Referring to the smaller size of conidia.

*Typus*: China, Shandong Province, Weihai city (122.17° E, 37.50° N), from intertidal sediment of a beach, 25 November 2020, M.M. Wang and Y. Zheng (HMAS 354890, holotype designated here, dried culture on PDA; culture ex-type CGMCC 3.22581 = WL05643; GenBank Accession No. ITS = PZ764957).

Pycnidia semi-immersed to superficial on oatmeal agar, solitary or clustered on sterilized toothpicks and pine needles. Mature pycnidia shiny black to charcoal-black, smooth or slightly glossy, hard; subglobose to globose 80–150 μm diam, some with an inconspicuous papillate ostiole. Pycnidial wall 4–6 cells thick, with outer cells dark brown, thick-walled, polygonal, pigmented; inner layers gradually becoming thin-walled and hyaline. Conidiogenous cells phialidic to subcylindrical, hyaline, smooth, 3.2–4.9 × 3.3–6.0 μm (mean 3.78 × 4.32 μm, *n* = 30), arising from the pycnidial wall. Conidia ellipsoidal to cylindrical, with rounded ends, 0.9–1.5 × 2.0–3.4 μm (mean 1.26 × 2.62 μm, *n* = 50), hyaline when released, becoming pale yellowish-brown at maturity, thin-walled, smooth.

*Culture characteristics*: Colonies on PDA are 45–50 mm diam in 7 d at 25 °C, flat, loose, nearly circular, margin entire, aerial mycelium orange white to pale orange (5A2–5A3), slightly raised in the center; reverse cream to orange white (4A2–5A2), nearly white at the margin, without obvious pigment diffusion. Colonies on OA are 50–55 mm diam, velvety, nearly circular, margin undulate, aerial mycelium white to light gray (1A1–6B2), denser in the center, sparser at the margin; reverse with concentric rings, white to butter yellow (1A1–4A5), without obvious pigment. Colonies on MEA are 45–55 mm diam, circular, loosely filamentous, margin slightly rough, aerial mycelium orange white to pale orange (6A2–6A3); reverse pale orange (6A3).

*Other examined isolates*: China, Shandong Province, Yantai city (121.29° E, 37.57° N), from intertidal sediment of a mudflat, 14 June 2014, X.M. Bian (WL03660).

*Notes*: Phylogenetically, *Paraconiothyrium microconidium* is closely related to *P. cyclothyrioides*, *P. iridis* and *P. salinum* (Figure 2). Morphologically, the new species can be recognized by the shape and size of the conidia: ellipsoidal to cylindrical, 0.9–1.5 × 2.0–3.4 μm in *P. microconidium* compared to short-cylindrical, 1–1.8 × 2.5–5 µm in *P. cyclothyrioides*, and short-cylindrical, 1–1.4 × 3.3–4 μm in *P. salinum* [3,12]. *Paraconiothyrium microconidium* and *P. iridis* cannot be compared morphologically, as *P. iridis* only produces a sexual morph in culture [37].

**Figure 7:**
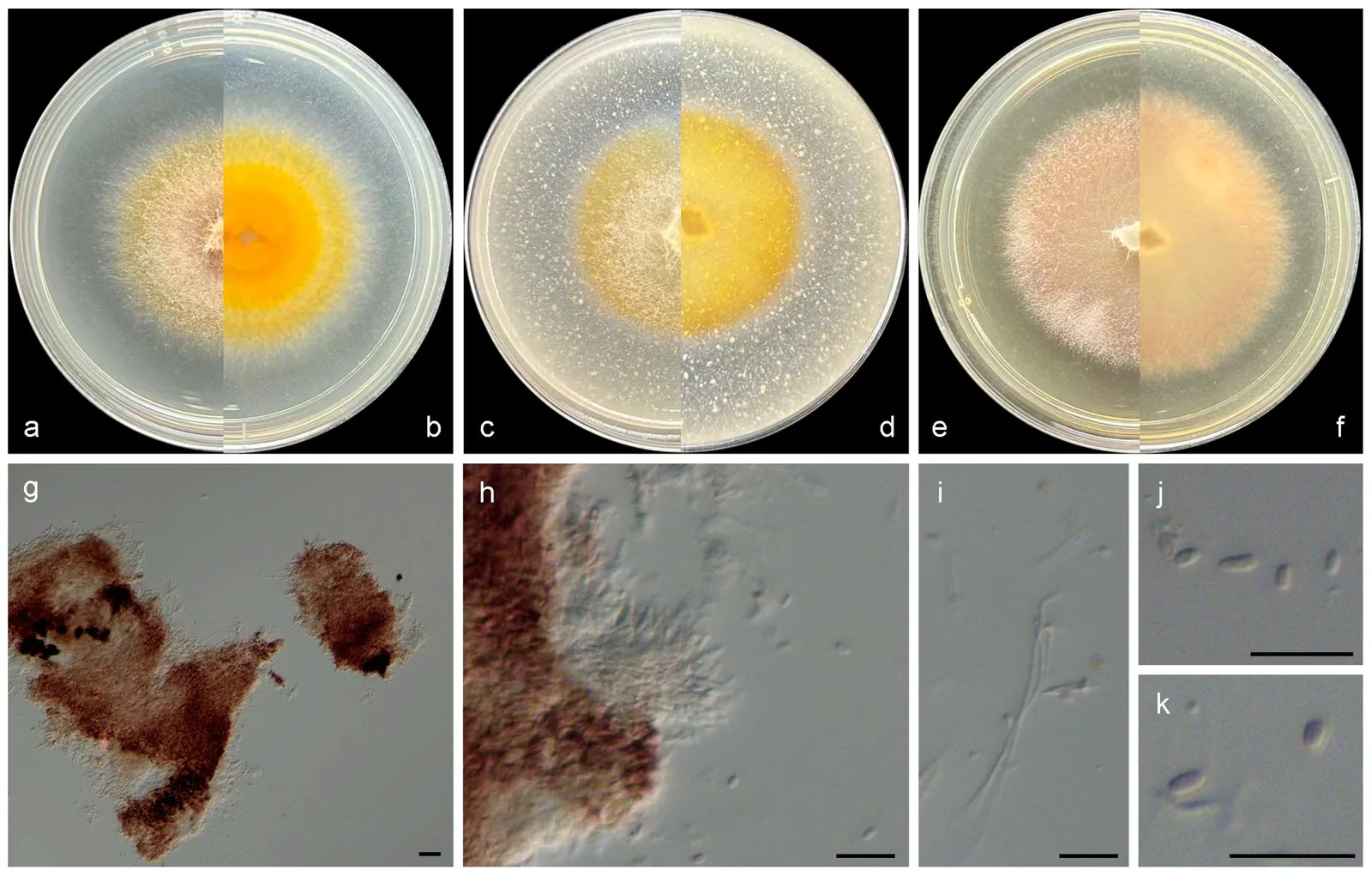
Morphological characteristics of *Paraconiothyrium qingdaoense* (from ex-type WL05883). (**a**–**f**): Colony morphology (obverse and reverse) on PDA, OA, and MEA after 7 days of cultivation at 25 °C. (**g**): Pycnidia. (**h**–**k**): Conidiogenous cells and conidia. Scale bars: (**g**,**h**) = 20 μm, (**i**–**k**) = 10 μm.

***Paraconiothyrium qingdaoense*** M.M. Wang, W.Y. Mo and W. Li sp. nov., Figure 7.

*FungalNames*: FN 573962

*Etymology*: Referring to the location of the type specimen of this new species, Qingdao city.

*Typus*: China, Shandong Province, Qingdao city (120.34° E, 36.06° N), from intertidal seawater of a beach, 12 March 2021, Y. Zheng, Z.H. Pan and Y.R. Ma (HMAS 354891, holotype designated here, dried culture on PDA; culture ex-type CGMCC 3.40480 = WL05883; GenBank Accession No. LSU = PZ764978, ITS = PZ764959).

Pycnidia irregular, pulvinate to pyriform, wall composed of multiple layers of dark brown to yellowish-brown, thick-walled angular cells, surface rough; mature pycnidia ca. 100–180 × 190–330 μm. Conidiogenous cells clustered on the inner pycnidial wall, phialidic, cylindrical to lageniform, 1.6–3.3 × 3.5–5.7 μm (mean 2.37 × 4.53 μm, *n* = 30), with a distinct apical conidiogenous neck. Conidia hyaline, aseptate, ellipsoidal to long-ellipsoidal, 1.4–2.4 × 1.7–3.7 μm (mean 1.78 × 2.57 μm, *n* = 50), with rounded ends, smooth-walled.

*Culture characteristics*: Colonies on PDA are 51–54 mm diam in 7 d at 25 °C, nearly circular, margin radiating, center creamy white to pale grayish-white (1A1–1B2), velvety, transitioning outward to bright orange-yellow to orange (5A6–6A7), forming a halo; reverse deep orange-yellow to yellowish-brown centrally (5A7–5C6), margin light yellow (2A4). Colonies on OA are 48–51 mm diam, nearly circular, center creamy white to pale grayish-white (1A1–1B2), surrounded by uniform light orange-yellow to orange (5A5–6A6); reverse uniformly deep orange-yellow to orange (5A7–6A7). Colonies on MEA are 45–48 mm diam., nearly circular, margin entire; center pale pinkish-brown to pale grayish-brown (9B3–4B3), transitioning to pale creamy-yellow to pale grayish-white; reverse uniformly pale pinkish-brown to pale yellowish-brown (9B3–4B4).

*Other examined isolates*: China, Guangdong Province, Shantou city, from intertidal sediment of a beach, 26 May 2014, W. Li and X.M. Bian (WL02530); Shandong Province, Qingdao city (120.34° E, 36.06° N), from intertidal seawater of a beach, 12 March 2021, Y. Zheng, Z.H. Pan and Y.R. Ma (WL07372); ibid. (WL07373).

*Notes*: Phylogenetically, *Paraconiothyrium qingdaoense* is closely related to *P. estuarinum* and *P. maculicutis* (Figure 2). Morphologically, the new species can be distinguished from the latter species by the shape and size of the conidia: ellipsoidal to long-ellipsoidal, 1.4–2.4 × 1.7–3.7 μm in *P. qingdaoense* compared to narrowly ellipsoidal to short-cylindrical, 1.4–2 × 3–6 µm in *P. estuarinum*, and ellipsoidal, 0.5–1.5 ×1.5–2.5 μm in *P. maculicutis* [3,36].

*Sporormiaceae* Munk, Dansk bot. Ark. 17(no. 1): 450 (1957)

*Westerdykella* Stolk, Trans. Br. mycol. Soc. 38(4): 422 (1955)

**Figure 8:**
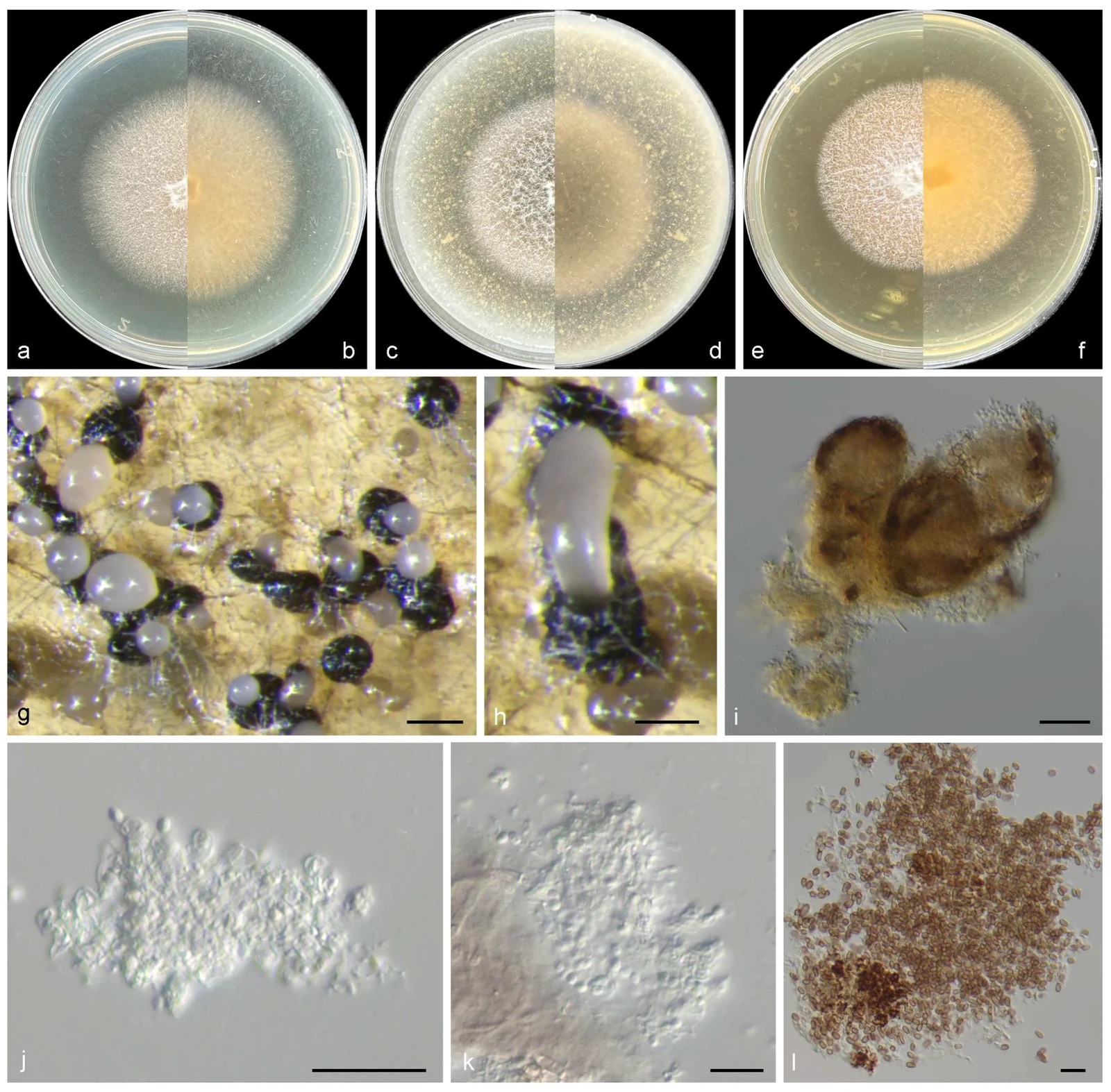
Morphological characteristics of *Westerdykella fulva* (from ex-type WL04732). (**a**–**f**): Colony morphology (obverse and reverse) on PDA, OA, and MEA after 7 days of cultivation at 25 °C. (**g**–**i**): Pycnidia. (**j**–**l**): Conidiogenous cells and conidia. Scale bars: (**g**,**h**) = 100 μm, (**i**) = 20 μm, (**j**–**l**) = 10 μm.

***Westerdykella fulva*** M.M. Wang, W.Y. Mo and W. Li sp. nov., Figure 8.

*FungalNames*: FN 573964

*Etymology*: Referring to the color of conidia, pale olive-brown.

*Typus*: China, the Yellow Sea (unknown longitude and latitude), from coastal sediment, 11 July 2019, Y.Y. Ma (HMAS 354892, holotype designated here, dried culture on PDA; culture ex-type CGMCC 3.40477 = WL04732; GenBank Accession No. ITS = PZ764963, rpb2 = PZ775730).

Pycnidia scattered or aggregated on toothpicks, pine needles, and agar, initially appearing as translucent to milky white globose protrusions, becoming black, carbonaceous, globose to subglobose at maturity, 80–120 μm diam, immersed or semi-immersed in the mycelial layer, partially protruding from the substrate surface, with white cirrus exuding from the ostiole at maturity; pycnidial wall composed of multiple layers of dark brown to black, thick-walled angular cells; wall surface smooth, with a distinct ostiole, cells around the ostiole slightly thickened. Conidiogenous cells arising from the inner pycnidial wall, phialidic or ampulliform, hyaline, 1.6–2.5 × 2.5–4.5 μm (mean 2.01 × 3.80 μm, *n* = 30), with a truncate apex and distinct conidiogenous scar. Conidia aseptate, hyaline when released, becoming pale olive-brown at maturity, ovoid or ellipsoidal, with rounded ends, smooth-walled, 1.3–2.3 × 1.8–3.7 μm (mean 1.74 × 2.46 μm, *n* = 50).

*Culture characteristics*: Ccolonies on PDA are 35–40 mm diam in 7 d at 25 °C, flat, floccose, nearly circular, margin entire; aerial mycelium reddish white (8A2); reverse pale orange (5A3). Colonies on OA are 25–35 mm diam, velvety, nearly circular, margin undulate, aerial mycelium white (1A1); reverse nearly white to light gray (1A1–1B1). Colonies on MEA are 30–35 mm diam, circular, loosely filamentous, margin slightly rough, aerial mycelium shell pink (8A3); reverse pale orange to light orange (6A3–6A4), with obvious pale brown pigment diffusion.

*Other examined isolates*: China, Shandong Province, Qingdao city (120.34° E, 36.06° N), from intertidal seawater of a beach, 12 March 2021, Y. Zheng, Z.H. Pan and Y.R. Ma (WL05882); the Yellow Sea, unknown location, collection date and collector (WL04439).

*Notes*: The genus *Westerdykella* was introduced in 1955, based on the type species, *W. ornata*, which was isolated from mangrove mud in Mozambique [5]. Currently, 14 *Westerdykella* species are listed in MycoBank (https://www.mycobank.org/; accessed on 10 July 2026). In this study, three new *Westerdykella* species were added. On the phylogenetic tree, *W. fulva* formed a single, well-support clade, closely related to *W. dispersa* and *W. sedimenticola* (Figure 3). Morphologically, this new species can be easily recognized from the color, shape and size of its conidia (hyaline to pale olive-brown, ovoid or ellipsoidal, 1.3–2.3 × 1.8–3.7 μm in *W. fulva* vs. hyaline, ellipsoidal, subglobose, some pyriform, 1.8–2.7 µm × 3.1–4.0 µm in *W. dispersa*, and hyaline, ovoid to ellipsoidal, 1.7–3.0 × 2.7–5.1 μm in *W. sedimenticola*) [38].

**Figure 9:**
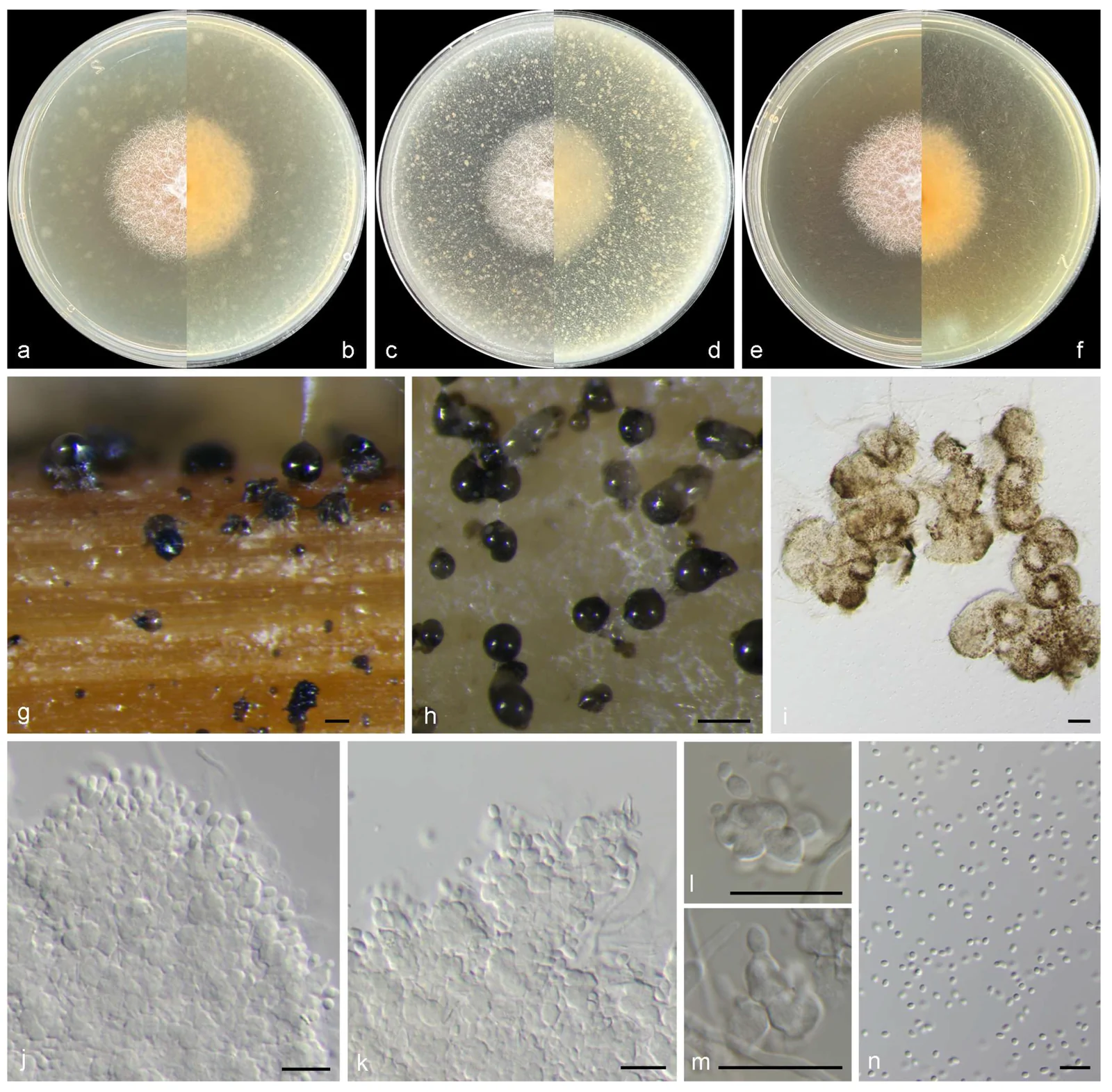
Morphological characteristics of *Westerdykella microcarpa* (from ex-type WL02396). (**a**–**f**): Colony morphology (obverse and reverse) on PDA, OA, and MEA after 7 days of cultivation at 25 °C. (**g**–**i**): Pycnidia. (**j**–**n**): Conidiogenous cells and conidia. Scale bars: (**g**,**h**) = 100 μm, (**i**) = 20 μm, (**j**–**n**) = 10 μm.

***Westerdykella microcarpa*** M.M. Wang, W.Y. Mo and W. Li sp. nov., Figure 9.

*FungalNames*: FN 573965

*Etymology*: Named after the smaller pycnidia of the new species.

*Typus*: China, Guangdong Province, Shantou city (116.76° E, 23.31° N), from intertidal sediment of a beach, 26 May 2014, W. Li and X.M. Bian (HMAS 354893, holotype designated here, dried culture on PDA; culture ex-type CGMCC 3.40476 = WL02396; GenBank Accession No. LSU = PZ764982, ITS = PZ764965, tef1 = PZ775710, rpb2 = PZ775732).

Pycnidia scattered or aggregated on toothpicks, pine needles, and agar, initially appearing as translucent to milky white globose protrusions, becoming black, globose to subglobose at maturity, 60–110 μm diam, immersed or semi-immersed in the mycelial layer, partially protruding from the substrate surface, with white cirrus exuding from the ostiole at maturity; pycnidial wall composed of multiple layers of dark brown to black, thick-walled angular cells; wall surface smooth, with a distinct ostiole, cells around the ostiole slightly thickened. Conidiogenous cells arising from the inner pycnidial wall, phialidic or ampulliform, hyaline, 2.0–3.5 × 3.1–4.8 μm (mean 2.75 × 3.91 μm, *n* = 30), with a truncate apex and distinct conidiogenous scar. Conidia aseptate, hyaline, ovoid, subglobose to globose, smooth-walled, 1.6–2.7 × 2.0–3.9 μm (mean 2.02 × 2.72 μm, *n* = 50).

*Culture characteristics*: Colonies on PDA are 35–40 mm diam in 7 d at 25 °C, flat, floccose, nearly circular, margin entire; aerial mycelium reddish white (8A2); reverse pale orange (5A3). Colonies on OA are 25–35 mm diam, velvety, nearly circular, margin undulate, aerial mycelium pale gray (1B1); reverse nearly white to light gray (1A1–1B1). Colonies on MEA are 30–35 mm diam, circular, loosely filamentous, margin slightly rough, aerial mycelium shell pink (8A3); reverse pale orange to light orange (6A3–6A4), with obvious pale brown pigment diffusion.

*Other examined isolates*: China, Guangdong Province, Shantou city (116.76° E, 23.31° N), from intertidal sediment of a beach, 26 May 2014, W. Li and X.M. Bian (WL07376); ibid. (WL07377).

*Notes*: Phylogenetically, *W. microcarpa* formed a single, well-supported clade, closely related to *W. capitulum* (Figure 3). Morphologically, this new species can be easily identified by the shape and size of conidia, which are ovoid, subglobose to globose, 1.6–2.7 × 2.0–3.9 μm in *W. microcarpa* compared to ellipsoidal, some globose, 3.0–4.1 µm × 2.4–3.4 µm in *W. capitulum* [38].

**Figure 10:**
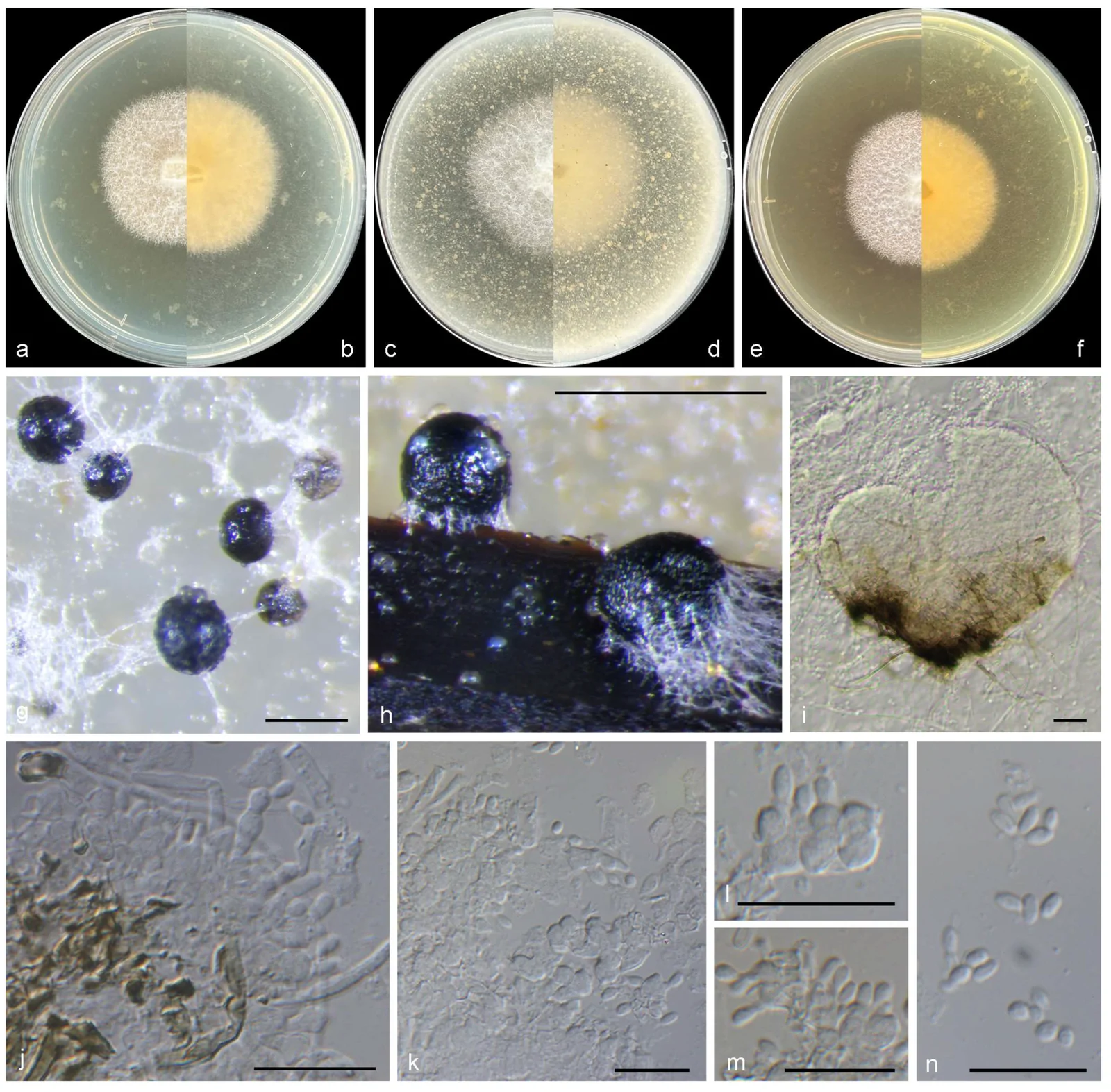
Morphological characteristics of *Westerdykella sedimenticola* (from ex-type WL05930). (**a**–**f**): Colony morphology (obverse and reverse) on PDA, OA, and MEA after 7 days of cultivation at 25 °C. (**g**–**i**): Pycnidia. (**j**–**n**): Conidiogenous cells and conidia. Scale bars: (**g**,**h**) = 500 μm, (**i**) = 20 μm, (**j**–**n**) = 10 μm.

***Westerdykella sedimenticola*** M.M. Wang, W.Y. Mo and W. Li sp. nov., Figure 10.

*FungalNames*: FN 573966

*Etymology*: Named after the habitat of the type specimen of this new species, sediment.

*Typus*: CHINA, Jiangsu Province, Yancheng city (119.88° E, 34.46° N), from intertidal sediment of a beach, 21 April 2021, Z.Q. Zeng and C. Liu (HMAS 354894, holotype designated here, dried culture on PDA; culture ex-type CGMCC 3.40481 = WL05930; GenBank Accession No. ITS = PZ764968, rpb2 = PZ775735).

Pycnidia scattered or aggregated on toothpicks, pine needles, and agar, initially appearing as translucent to milky white globose protrusions, becoming black, carbonaceous, globose to subglobose at maturity, 60–80 μm diam, immersed or semi-immersed in the mycelial layer, partially protruding from the substrate surface, with white cirrus exuding from the ostiole at maturity; pycnidial wall composed of multiple layers of dark brown to black, thick-walled angular cells; wall surface smooth, with a distinct ostiole, cells around the ostiole slightly thickened. Conidiogenous cells arising from the inner pycnidial wall, phialidic or ampulliform, hyaline, 1.8–3.6 × 2.5–4.1 μm (mean 1.59 × 3.35 μm, *n* = 30), with a truncate apex and distinct conidiogenous scar. Conidia aseptate, hyaline, ovoid to ellipsoidal, with rounded ends, smooth-walled, 1.7–3.0 × 2.7–5.1 μm (mean 2.28 × 3.66 μm, *n* = 50).

*Culture characteristics*: Colonies on PDA are 30–35 mm diam in 7 d at 25 °C, flat, floccose, nearly circular, margin entire; aerial mycelium orange white (6A2); reverse pale orange to light orange (5A3–5A4). Colonies on OA are 30–35 mm diam, velvety, nearly circular, margin undulate, aerial mycelium white (1A1); reverse nearly white to pale orange (5A3). Colonies on MEA are 30–35 mm diam, circular, loosely filamentous, margin slightly rough, aerial mycelium reddish white (8A2); reverse pale orange (6A3), with obvious pale brown pigment diffusion.

*Other examined isolates*: CHINA, Jiangsu Province, Yancheng city (119.88° E, 34.46° N), from intertidal sediment of a beach, 21 April 2021, Z.Q. Zeng and C. Liu (WL07374); ibid. (WL07375).

*Notes*: See “notes” in *W. fulva*.

## 4. Discussion

The seven new species described in this study were delineated based on distinct morphological features and phylogenetic analyses, in accordance with modern integrative taxonomy principles [39,40]. Notably, the identification of a new *Montagnula* species based solely on its asexual morph is significant, given that most members of this genus, including the type species *M. infernalis*, are only known from their sexual states. Only four species have previously been reported to possess an asexual morph [9,30]. Similarly, the three new *Westerdykella* species are characterized by their pycnidial asexual morph. This feature is rarely documented for a genus that is primarily defined by its cleistothecial sexual morph with multi-spored asci [5,9,10]. Our results, therefore, reinforce the value of asexual morph characters in defining species boundaries within these genera. The three new *Paraconiothyrium* species can easily be distinguished from other known species, particularly the most similar ones *(P. estuarinum*, *P. cyclothyrioides*, and *P. maculicutis*) thanks to their unique combination of pycnidial texture (e.g., hard and carbonaceous), unusual conidiomatal morphology (e.g., irregular and pulvinate), and significantly smaller conidial and conidiogenous cell dimensions [3,7,41]. The high support values observed in our multi-locus phylogenetic analyses (LSU, ITS, tef1, and rpb2) provide robust molecular evidence for the novelty of all seven taxa, corroborating the morphological observations. Our findings will enhance the ongoing taxonomic discussions within these genera.

The new *Montagnula* species described here can be compared with the controversial *M. donacina*. While recent studies have reduced several species, including *M. chromolaenicola*, *M. puerensis*, *M. saikhuensis*, and *M. thailandica*, to synonyms of *M. donacina* [42], other research has retained them as distinct species [2]. Our new species, with its distinct morphology and marine origin, highlight the need for careful molecular and morphological scrutiny to resolve species boundaries within *Montagnula*. The paraphyletic nature of *Paraconiothyrium* remains a challenge to the taxonomy of the *Didymosphaeriaceae* family [7,34]. The three novel species described here further expand the morphological and ecological diversity of the genus. Future studies incorporating genomic data are necessary to determine whether a formal reclassification of *Paraconiothyrium* into monophyletic units is warranted. Describing these three new *Westerdykella* species based solely on asexual morphs reveals a gap in our understanding of the genus. Particularly, many species are only known from their sexual morph (e.g., *W. ornata*, *W. purpurea*, *W. aquatica*, and *W. reniformis*) [10,11], suggesting that the full diversity and life cycles of *Westerdykella* are still poorly understood [9].

A striking aspect of this study is the marine origin of all seven novel species. Although *Montagnula*, *Paraconiothyrium*, and *Westerdykella* are ecologically versatile and have been found on various substrates around the world [2,3], their recent discovery in hypersaline and marine environments suggests that they have a broader ecological amplitude than was previously thought. For instance, *Montagnula* species are increasingly being reported in saline habitats, including *M. globospora* and *M. terricola* in Spanish hypersaline lagoons [9]. *Paraconiothyrium* has recently been found on marine macroalgae with *P. salinum* [12]. *Westerdykella dispersa* is a common marine-derived species that has been isolated from diverse substrates and is known for producing various bioactive compounds [43]. The unique morphological features of our new species, such as the hard carbonaceous pycnidia of the *Paraconiothyrium* species and the white cirri exuding from the ostioles of the *Westerdykella* species, may represent adaptations to the marine environment, such as protection from desiccation or enhanced spore dispersal. Since *Westerdykella* species are important sources of enzymes (e.g., phytase) [10] and antibiotics (e.g., melanocidin IV and chetracin B) [11], and *Paraconiothyrium* species are known mycoparasites [3], these marine isolates could harbor novel biological activities worthy of further investigation.

This study expands the known geographical and ecological distribution of all three genera. *Montagnula* species, which have been recorded in numerous countries across multiple continents [2], have now been documented in new marine substrates. *Paraconiothyrium* species have a global distribution on diverse plant hosts [3,8] and have been infrequently reported in marine habitats. Our new species adds to this list, alongside *P. salinum* [12]. The three new *Westerdykella* species in marine sediments expands the number of species identified in these environments, alongside *W. dispersa*, *W. purpurea*, and *W. ornata* [5,44,45]. While our species did not exhibit strict habitat specificity, their consistent isolation from marine substrates suggests a potential preference for, or adaptation to, these saline conditions, potentially representing an ecological shift from the predominantly terrestrial or soil-dwelling habits of their congeners. Collectively, these findings underscore the need for more extensive sampling, particularly of marine sediments and flora, to fully understand the global diversity and distribution patterns of these genera [46,47].

## Figures and Tables

**Table 1:** The sequences of the new and reference pleosporalean strains examined in this study.

Species	Isolate	GenBank Accession No.			
		LSU	ITS	tef1	rpb2
***Didymosphaeriaceae***					
*Montagnula acaciae*	MFLUCC 19-0087 T	ON117299	ON117281	ON158094	--
*M. agaves*	ZHKUCC 22-0123 T	OR198820	OR198819	OR195064	--
*M. aloes*	CBS 132531 T	NG_042676	NR_111757	--	--
*M. appendiculata*	CBS 109027 T	AY772016	DQ435529	--	--
*M. aquatica*	MFLU 22-0171 T	OP605986	OP605992	--	--
*M. aquilariae*	KUNCC 22-10815T	OP482265	OP452927	OP426318	--
	KUNCC 23-14430	OR583119	OR583100	OR588091	OR588110
*M. bellevaliae*	MFLUCC 14-0924T	KT443902	KT443906	KX949743	--
*M. camporesii*	MFLUCC 16-1369 T	MN401742	MN401746	MN397908	MN397909
*M. chiangraiensis*	MFLUCC 17-1421 T	NG_068707	MT214349	--	--
*M. chromolaenae*	MFLUCC 17-1435T	NG_068708	NR_168865	--	--
*M. chromolaenicola*	MFLUCC 17-1469T	NG_070948	NR_168866	MT235773	MT235809
*M. cirsii*	MFLUCC 13-0680 T	KX274249	KX274242	KX284707	--
*M. coffeae*	KUNCC 24-18337 T	OR428421	OR428399	OR509753	--
*M. cylindrospora*	CBS 146572 T	LN907351	LT796834	LT797074	LT796994
*M. donacina*	KUNCC 23-14428	OR583124	OR583105	OR588096	OR588115
*M. globospora*	FMR 20550 T	PP973382	PP973384	PP973931	--
*M. graminícola*	MFLUCC 13-0352 T	KM658315	KM658314	--	--
*M. guiyangensis*	HKAS 124556 T	OP600484	OP605989	--	--
*M. jonesii*	MFLUCC 16-1448 T	KY273276	KY313619	KY313620	--
*M. krabiensis*	MFLUCC 16-0250 T	NG068826	NR168179	MH412776	--
*M. lijiangensis*	HKAS 126541 T	OR583127	OR583108	OR588099	OR588118
*M. menglaensis*	KUNCC 23-14424 T	OR583130	OR583111	OR588102	OR588121
***M. minispora***	**WL07369 = CGMCC 3.40482 T**	**PZ764971**	**PZ764947**	**PZ775706**	**PZ775718**
	**WL07370**	**PZ764972**	**PZ764948**	**PZ775707**	**PZ775719**
	**WL07371**	**PZ764973**	**PZ764949**	**PZ775708**	**PZ775720**
*M. opulenta*	UTHSC:DI16-208 T	LN907351	LT796834	LT797074	--
*M. puerensis*	KUMCC 20-0225 T	MW575866	MW567739	MW573959	--
*M. saikhuensis*	MFLUCC 16-0315 T	KU743210	KU743209	--	--
*M. scabiosae*	MFLUCC 14-954 T	NG_059602	NR_155378	--	--
*M. shangrilana*	KUNCC 23-14434 T	OR583132	OR583113	OR588104	OR588123
*M. sinensis*	GMBCC 1185 T	PX091389	PQ346644	PX101340	PX101322
*M. striatispora*	CBS 385.65 T	NG_064048	MH858624	--	--
*M. terricola*	CBS 100256 T	PP973380	OQ708961	PP973929	--
*M. thailandica*	MFLUCC 17-1508 T	MT214446	MT214352	MT235774	MT235810
*M. thevetiae*	HKAS 126964 T	OR583134	OR583115	OR588106	OR588125
*M. verniciae*	CGMCC 3.24435 T	OR253273	OR269139	OR251166	--
*M. yunnanensis*	GMBCC 1174 T	PX091400	PQ346645	PX101351	PX101333
*Neokalmusia jonahhulmei*	KUMCC 21-0818 T	ON007039	ON007043	ON009133	ON009137
*Paraconiothyrium africanum*	CBS 121166 T	NG_069117	EU295650	--	--
*P. ajrekarii*	NFCCI 4810	MT372905	MT372906	--	--
*P. archidendri*	MFLUCC 17-2429	MK347974	MK347757	MK360044	--
	CBS 168.77	JX496162	JX496049	--	--
*P. babiogorense*	CBS 128292 T	MH876291	MH864845	--	--
*P. bishopiae*	BRIP 72437b T	OP598066	OP599629	--	--
*P. brasiliense*	CBS 100299 T	JX496124	AY642531	--	--
*P. camelliae*	NTUCC 18-096	MT071269	MT112293	MT277330	--
*P. cyclothyrioides*	CBS 972.95 T	JX496232	JX496119	--	--
***P. ellipsosporum***	**WL02373**	**PZ764974**	**PZ764950**	**PZ775709**	**PZ775721**
	**WL04294**	**--**	**PZ764951**	**--**	**PZ775722**
	**WL05874 = CGMCC 3.40479 T**	**PZ764975**	**PZ764952**	**PZ775713**	**PZ775723**
	**WL05908**	**--**	**PZ764953**	**--**	**PZ775724**
	**WL06131**	**PZ764976**	**PZ764954**	**PZ775715**	**--**
	**WL06137**	**--**	**PZ764955**	**--**	**PZ775725**
*P. estuarinum*	CBS 109850 T	JX496129	JX496016	--	--
*P. fuckelii*	CBS 797.95	GU237960	JX496113	--	--
*P. fungicola*	CBS 113269 T	JX496133	JX496020	--	--
*P. fuscomaculans*	CBS 116.16	MH866170	MH854649	--	--
*P. hakeae*	CBS 142521 T	KY979809	KY979754	--	KY979847
*P. hawaiiense*	CBS 120025 T	NG_070608	DQ885897	--	--
*P. iridis*	CBS 146036	MT223919	MT223827	--	MT223695
*P. kevinhydei*	CGMCC 3.27707 T	PQ184711	PQ249235	PQ346511	PQ379995
*P. lini*	CBS 253.92	GU238093	--	--	--
*P. lycopodinum*	CBS 134705 T	NG_058029	--	--	--
*P. maculicutis*	CBS 101461	EU754200	--	--	--
*P. magnoliae*	MFLUCC 10-0278	KJ939283	KJ939280	--	--
***P. microconidium***	**WL03660**	**--**	**PZ764956**	**--**	**--**
	**WL05643 = CGMCC 3.22581 T**	**--**	**PZ764957**	**--**	**--**
*P. nelloi*	MFLU 14-0813 T	KP711365	KP711360	--	--
*P. polonense*	CBS:134153 T	KF700360	--	--	--
***P. qingdaoense***	**WL02530**	**PZ764977**	**PZ764958**	**PZ775711**	**--**
	**WL05883 = CGMCC 3.40480 T**	**PZ764978**	**PZ764959**	**--**	**PZ775726**
	**WL07372**	**PZ764979**	**PZ764960**	**--**	**PZ775727**
	**WL07373**	**PZ764980**	**PZ764961**	**--**	**PZ775728**
*P. rosae*	MFLU 15-1115 T	MG829041	MG828932	--	--
*P. salinum*	CMG 49 T	--	MN369540	MN380481	--
*P. thysanolaenae*	MFLUCC 10-0550 T	KP744496	KP744453	--	--
*P. tiliae*	CBS 265.94	EU754139	--	--	--
*P. variabile*	CBS 121163 T	--	EU295639	--	--
*P. yunnanensis*	ZHKUCC 22-0196 T	OP297767	OP297797	OP321566	OP321560
***Sporormiaceae***					
*Westerdykella aquatica*	JAUCC 1788 T	MH411090	MH411093	--	--
*W. canariensis*	CBS 151789 T	OZ199754	OZ199753	OZ199786	OZ199785
*W. capitulum*	CBS 337.65 IT	MH870234.1	MH858595.1	--	--
*W. centenaria*	CBS 142400 T	NG_058468	NR_156002	--	--
*W. cylindrica*	CBS 454.72 T	AY004343	AY943056	DQ497610	--
*W. dispersa*	CBS 297.56 T	MH869191	MH857647	--	--
*W. formosana*	NTUPPMCC 22-255 T	PV476875	PV476837	--	--
***W. fulva***	**WL04439**	**--**	**PZ764962**	**PZ775712**	**PZ775729**
	**WL04732 = CGMCC 3.40477 T**	**--**	**PZ764963**	**--**	**PZ775730**
	**WL05882**	**PZ764981**	**PZ764964**	**PZ775714**	**PZ775731**
*W. globosa*	IMI 082625 T	--	AY943046	--	--
*W. globosa*	NTUPPMCC 22-245	PV426323	PV476849	--	--
***W. microcarpa***	**WL02396 = CGMCC 3.40476 T**	**PZ764982**	**PZ764965**	**PZ775710**	**PZ775732**
	**WL07376**	**PZ764983**	**PZ764966**	**PZ775716**	**PZ775733**
	**WL07377**	**PZ764984**	**PZ764967**	**PZ775717**	**PZ775734**
*W. minutispora*	CBS 338.65T	MH870235	MH858596	--	--
*W. multispora*	CBS 383.69	GQ203754	GQ203799	--	--
*W. nigra*	CBS 416.72	GQ203755	GQ203800	--	--
*W. ornata*	CBS 379.55 T	GU301880	MH857522	GU349021	GU371803
*W. purpurea*	NTUPPMCC 22-252	PV426326	PV476850	--	--
*W. reniformis*	DAOM 242243 T	JX235704	JX235700	--	--
***W. sedimenticola***	**WL05930 = CGMCC 3.40481 T**	**--**	**PZ764968**	**--**	**PZ775735**
	**WL07374**	**--**	**PZ764969**	**--**	**PZ775736**
	**WL07375**	**--**	**PZ764970**	**--**	**PZ775737**

Notes: T = ex-type, IT = ex-isotype. Sequences newly generated in this study are marked in bold.

## Data Availability

All sequences generated in this study were submitted to GenBank (https://www.ncbi.nlm.nih.gov; accessed on 15 July 2026).
